# Cyclops lesion – The entity causing loss of knee extension after ACL reconstruction surgery: A case report

**DOI:** 10.1016/j.ijscr.2021.106554

**Published:** 2021-11-02

**Authors:** Charanjeet Singh, Shamala Devi Vellasamy, Jessica Fiolin, Sholahuddin Rhatomy

**Affiliations:** a Department of Orthopaedic & Trauma Surgery, Gleneagles Hospital Kuala Lumpur, Malaysia; b Jakarta Knee, Shoulder and Orthopaedic Sport Clinic, Pondok Indah Hospital, Jl. Metro Duta Kav UE, Jakarta 12310, Indonesia; c Department of Orthopaedic and Traumatology, Soeradji Tirtonegoro Hospital, Klaten, Faculty of Medicine, Public Health of Nursing, Universitas Gadjah Mada, Yogyakarta, Indonesia

**Keywords:** Cyclops lesion, ACL reconstruction, Loss of extension

## Abstract

Cyclops is a fibrous nodule on the tibial side of the knee and it is one of the common complications that arises after anterior cruciate ligament (ACL) reconstruction surgery that causes loss of knee extension. In this literature, we have discussed about a young gentleman who presented with unresolved swelling and inability to extend knee joint fully 1 year after ACL reconstruction surgery. Cyclops lesion was identified by clinical examination and magnetic resonance imaging (MRI). An arthroscopic excision of the cyclops lesion was successfully done on this patient which resulted in a resolution of swelling and progressive improvement of knee extension.

## Highlights

• Anterior cruciate ligament (ACL) reconstruction is one of the most common procedures performed in the knee.
• Cyclops lesion is one of the most common complications causing loss of extension after an ACLR.
• Treatment requires arthroscopic excision of the lesion and aggressive rehabilitation.
• Although prognosis is good, prompt diagnosis and hence treatment should be performed correctly.

## 1 Introduction

Anterior cruciate ligament (ACL) is one of the most important ligament that consist of a band of dense connective tissue which courses from the femur to the tibia. It functions mainly for mechanical support of the knee. Rupture of ACL is one of the most common ligament injuries to the knee. About more than 100,000 ACL reconstruction surgeries are performed each year in the United States [1]. The principal aim of this reconstructive surgery is to restore the function and stability of the knee joint after an injury. However, as in every other surgery, this ACL reconstructive surgery also has its own complications. Common complications of ACL reconstructive surgery can be classified into intraoperative and post-operative complications. Intraoperative complications are malpositioned tunnels, improper tensioning and failure of graft fixation. Post-operative complications include infection, arthrofibrosis, graft failure, loss of extension and osteoarthritis [2].

Cyclops lesion is an ovoid-shaped mass formed following an ACL injury with or without reconstruction [3]. A recent literature review elaborated the risk factors of developing cyclops lesion include female gender, greater graft volume, bony avulsion injuries, excessively anterior tibial tunnel, and double-bundle ACLR [2].

Cyclops lesions usually found 10–25% after ACL reconstruction [4]. Although typically formed within the first six months after surgery, its size typically remains constant in size over two years. Cyclops lesion which causes a loss of terminal extension, anterior knee pain and altered gait after an ACL reconstruction is called cyclops syndrome [5].

Management of cyclops syndrome requires arthroscopic excision followed by intensive physiotherapy to regain knee extension [2]. We reported a case of cyclops treated successfully with arthroscopic excision and physiotherapy which showed good to excellent result. This paper was written according to SCARE guideline [6].

## 2 Case presentation

**Fig. 1:**
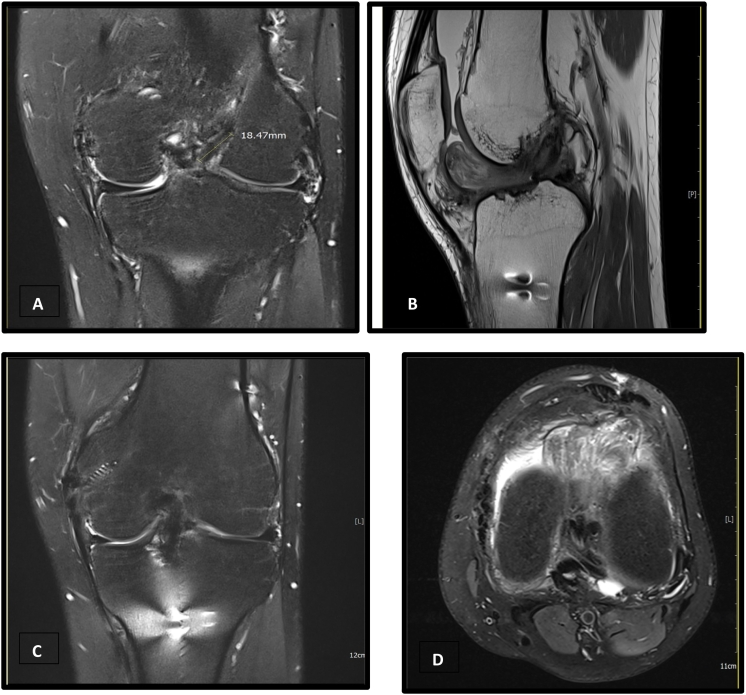
A–D, MRI examination showing cyclops lesion. A, MRI coronal T2 weighted image showing cyclops lesion size of 18.47 mm; B, medial extension of cyclops lesion; C, MRI showed intact ACL with MCL repair seen; D, axial view showing position of cyclops lesion at the intercondylar notch.

A 26 years old male patient had undergone ACL reconstruction (Patellar Tendon Bone) with MCL repair on 21st June 2019. After 4 months (31st October 2019), he presented with swelling and stiffness of knee joint for the past 2 months. However, the swelling persisted for 7 months after the ACL reconstruction surgery. MRI (Fig. 1A–C) was done to evaluate the cause of knee stiffness and persistent swelling. A heterogenous intermediate signal lesion in the anterior intercondylar notch abutting Hoffa's fat pad likely cyclops lesion was found. The ACL and MCL reconstruction appear satisfactory in the MRI. There was also chronic sprain of fibular collateral ligament and popliteal tendon noted on the MRI.

**Fig. 2:**
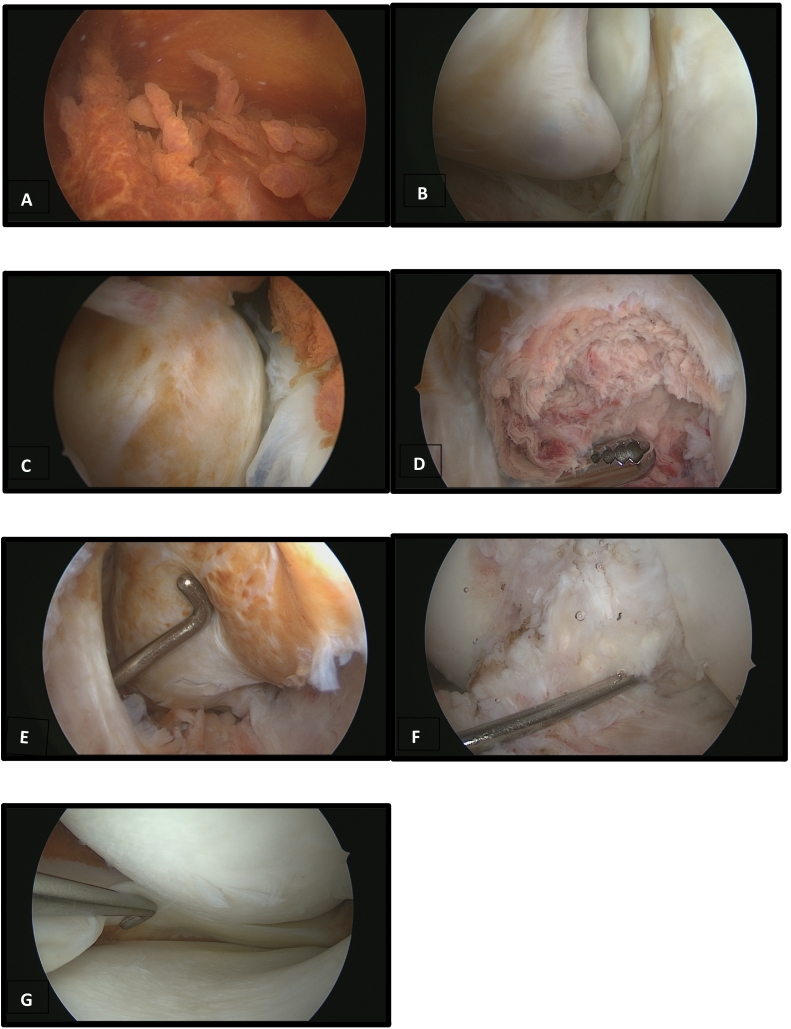
A, Arthroscopic image showing synovitis of the knee; B, cyclops lesion at the intercondylar notch; C, well surrounded cyclops with synovitis; D, granulation tissue once cyclops is opened; E, frontal view of cyclops lesion; F, intact ACL after cyclops excision; G, intact medial meniscus with chondral frame.

On arthroscopy, the lesion appears as a head soft tissue mass with a focal area of reddish discoloration (Fig. 2A–G). Sample specimen was sent to laboratory and histopathological examination showed fibrocartilaginous tissue. Based on clinical examination assessment, radiological, arthroscopic features, and histopathological examination, cyclops lesion on left knee was made as a definite diagnosis for this patient. He was advised to undergo arthroscopic excision to remove the cyclops lesion. After the excision, a routine post op physiotherapy was done by the patient to mobilize the knee joint. Patient regained full extension within 3 months of follow up and patient was very satisfied with the result.

## 3 Discussion

Cyclops lesion is also known as focal anterior arthrofibrosis usually located in the intercondylar notch close to the tibial insertion of anterior cruciate ligament. The cyclops lesion got its name as such due to its appearance in arthroscopy as a bulbous head like appearance with areas of reddish bluish discoloration [7]. It is made up of central granulation tissue surrounded by dense fibrous tissue. In the early stages the lesion may appear as a fibrosis tissue. However, it may evolve to stage fibrocartilaginous soft tissue in the late stages [8]. The origin of cyclops lesion is multifactorial as it can be from the fat metaplasia of infrapatellar, fibrosis of the intracondylar, debris from drilling the tibial tunnel, residual tibial ACL stump or the graft itself.

The distinctive and common sign of the presence of cyclops lesion is inability to extend the knee fully and unresolving swelling following an ACL reconstruction surgery. It usually occurs 2–3 months after the surgery however it is possible to occur after years of surgery depending on different individual. The loss of knee extension is generally about 10 degrees but it is sufficient to disrupt the patient's daily routine. An audible clunk sound, quadriceps dysfunction and pain at the anterior region of knee is also usually associated with cyclops lesion [9].

Cyclops nodule formation is a one of the significant complication after ACL reconstruction surgery. The incidence of post-operative cyclops nodule formation ranges from 1.9 to 10.6% [9], [10], [11]. The cyclops lesion was first described by Jackson and Schaefer in 1990 in patients who have done ACL reconstruction surgery [3]. They presented with loss of full extension with development of an audible and palpable “clunk” sound with terminal extension. The term cyclops was derived from Greek mythology where the cyclops lesion's appearance in arthroscopy as a soft-tissue nodule with surface vessels resembling the eye of cyclops.

## 4 Conclusion

In conclusion, it is essential to identify the underlying cause of loss of knee extension especially cyclops lesion in any ACL injury patient as it is easily treatable by doing arthroscopic resection and it will also result in satisfactory post-op outcome.

## Ethical approval

This ethical approval from Medical and Health Research Ethics Committee, Faculty of Medicine, Public Health and Nursing, Universitas Gadjah Mada.

## Funding

No source of funding.

## CRediT authorship contribution statement

Charanjeet Singh, Shamala Devi Vellasamy,J essica Fiolin, Sholahuddin Rhatomy:

- conceived the study.

- collected data.

- analysed data.

- prepared and drafted the manuscript, edited manuscript and reviewed the manuscript.

## Guarantor

Sholahuddin Rhatomy.

## Registration of research studies

1. Name of the registry: research registry
2. Unique identifying number or registration ID: researchregistry7269
3. Hyperlink to your specific registration (must be publicly accessible and will be checked): https://researchregistry.knack.com/researchregistry#home/registrationdetails/616be2e33d85d70020bc421b/

## Consent

Written informed consent was obtained from the patient for publication of this case report and accompanying images. A copy of written consent is available for review by Editor-in-Chief of this journal on request.

## Provenance and peer review

Not commissioned, externally peer-reviewed.

## Declaration of competing interest

The authors declare no conflict of interest.
